# Fidelity of Delivery and Contextual Factors Influencing Children’s Level of Engagement: Process Evaluation of the Online Remote Behavioral Intervention for Tics Trial

**DOI:** 10.2196/25470

**Published:** 2021-06-21

**Authors:** Kareem Khan, Chris Hollis, Charlotte L Hall, Elizabeth Murray, E Bethan Davies, Per Andrén, David Mataix-Cols, Tara Murphy, Cris Glazebrook

**Affiliations:** 1 Division of Psychiatry and Applied Psychology School of Medicine University of Nottingham Nottingham United Kingdom; 2 NIHR MindTech Medtech Co-operative Institute of Mental Health University of Nottingham Nottingham United Kingdom; 3 NIHR Nottingham Biomedical Research Centre Institute of Mental Health University of Nottingham Nottingham United Kingdom; 4 Research Department of Primary Care and Population Health University College London London United Kingdom; 5 Centre for Psychiatry Research Department of Clinical Neuroscience, Karolinska Institutet Stockholm Health Care Services, Stockholm County Council Stockholm Sweden; 6 Tic Disorder Clinic Great Ormond Street Hospital for Children NHS Foundation Trust London United Kingdom

**Keywords:** process evaluation, implementation fidelity, Tourette syndrome, chronic tic disorders, online behavioral intervention, mixed methods, children and young people

## Abstract

**Background:**

The Online Remote Behavioral Intervention for Tics (ORBIT) study was a multicenter randomized controlled trial of a complex intervention that consisted of a web-based behavioral intervention for children and young people with tic disorders. In the first part of a two-stage process evaluation, we conducted a mixed methods study exploring the reach, dose, and fidelity of the intervention and contextual factors influencing engagement.

**Objective:**

This study aims to explore the fidelity of delivery and contextual factors underpinning the ORBIT trial.

**Methods:**

Baseline study data and intervention usage metrics from participants in the intervention arm were used as quantitative implementation data (N=112). The experiences of being in the intervention were explored through semistructured interviews with children (n=20) and parent participants (n=20), therapists (n=4), and referring clinicians (n=6). A principal component analysis was used to create a comprehensive, composite measure of children and young people’s engagement with the intervention. Engagement factor scores reflected relative uptake as assessed by a range of usage indices, including chapters accessed, number of pages visited, and number of log-ins. The engagement factor score was used as the dependent variable in a multiple linear regression analysis with various contextual variables as independent variables to assess if there were any significant predictors of engagement.

**Results:**

The intervention was implemented with high fidelity, and participants deemed the intervention acceptable and satisfactory. The engagement was high, with child participants completing an average of 7.5 of 10 (SD 2.7) chapters, and 88.4% (99/112) of participants completed the minimum of the first four chapters—the predefined threshold effective dose. Compared with the total population of children with tic disorders, participants in the sample tended to have more educated parents and lived in more economically advantaged areas; however, socioeconomic factors were not related to engagement factor scores. Factors associated with higher engagement factor scores included participants enrolled at the London site versus the Nottingham site (*P*=.01), self-referred versus clinic referred (*P*=.04), higher parental engagement as evidenced by the number of parental chapters completed (n=111; ρ=0.73; *P*<.001), and more therapist time for parents (n=111; ρ=0.46; *P*<.001). A multiple linear regression indicated that parents’ chapter completion (β=.69; *t*_110_=10.18; *P*<.001) and therapist time for parents (β=.19; *t*_110_=2.95; *P*=.004) were the only significant independent predictors of child engagement factor scores.

**Conclusions:**

Overall, the intervention had high fidelity of delivery and was evaluated positively by participants, although reach may have been constrained by the nature of the randomized controlled trial. Parental engagement and therapist time for parents were strong predictors of intervention implementation, which has important implications for designing and implementing digital therapeutic interventions in child and adolescent mental health services.

**International Registered Report Identifier (IRRID):**

RR2-10.1186/s13063-019-3974-3

## Introduction

Tics are sudden, brief, rapid, and recurrent nonrhythmic movements or vocalizations that are more common in children and young people (CYP) than in adults [1] and more prevalent in males than in females [2]. Tic onset typically occurs between the ages of 3 and 8 years (mean onset is 6 and 7 years of age) [3], with the reported average age of greatest tic severity by the age of 10 years [4]. Although most CYP with tics only require educational support as the main form of treatment [5], there are interventions available for severe or disabling tics, as in Tourette syndrome or chronic tic disorders. Historically, pharmacotherapy, such as antipsychotics, has been the first line of treatment for severe tics; however, they often have undesirable side effects, such as weight gain and sleepiness [6]. Behavioral interventions are appealing and effective alternatives to pharmacotherapy. However, they require the patient to invest time and energy in practicing demanding behavioral techniques, such as tic control or habit reversal. Despite the benefits and evidence-based effectiveness of behavioral therapies for tic disorders [7-9], there is great difficulty in patients accessing behavioral treatments because of a shortage of trained therapists [10]. A promising development in increasing accessibility to behavioral treatments is the use of digital health interventions (DHIs) [11]. Preliminary evidence suggests that DHIs are efficacious for CYP with tic disorders in pilot randomized controlled trials (RCTs) [12-14]. A study that has assessed DHIs for tic disorders is the *Online Remote Behavioral Intervention for Tics* (ORBIT) trial, which has been described in detail previously [15] (see Textbox 1 for a brief description).

**Brief description of the ORBIT (Online Remote Behavioral Intervention for Tics) trial.**

**Design:** A 10-week, 2-armed, parallel-group, single-blind randomized controlled trial with an embedded process evaluation.

**Aim:** To evaluate the effectiveness of a web-based, remote, therapist-supported, and parent-guided behavioral intervention for tics, initially developed and piloted in Sweden.

**Intervention group:** A total of 112 children and young people received 10 modules (called "chapters") of behavioral therapy following the principles of exposure and response prevention via a secure web-based platform, with access to a therapist, delivered over a period of 10-12 weeks.

**Control group:** A total of 112 children and young people received 10 chapters of psychoeducation via a secure web-based platform, with access to a therapist, delivered over a period of 10-12 weeks.

**Primary outcome:** Total Tic Severity Score on the Yale Global Tic Severity Scale at 3 months postrandomization.

**Therapist role:** Both children and parents had regular contact with a therapist during the 10-12 week period via messages that were sent within the treatment platform (resembling an email) or via telephone, if required. The therapist was also able to directly comment on exercises that the participant had been working on and give specific feedback to motivate participants.

**Parent role:** One or both of the child's parents received a separate log-in to the web-based treatment platform, where they could access their own chapters. The parent chapters contained information regarding parent coping strategies and how to support their child. The parents also had access to the assigned therapist.

The population impact of any given intervention depends on both its effectiveness and its reach, defined as the proportion of the target population who access the intervention [16]. Although RCTs are the *gold standard* method for determining efficacy, additional data are needed before deciding whether an intervention should be adopted into mainstream health care. These additional data include understanding the reach of the intervention and the extent to which the data from an RCT, where the delivery of the intervention is often tightly controlled and monitored, can be extrapolated to use in routine health care. It has been argued that studies addressing questions about reach and effectiveness in routine care are needed [17,18]. However, such studies are expensive, and a process evaluation conducted alongside an RCT is an efficient method of maximizing the information yielded by the trial.

The Medical Research Council (MRC) has developed specific guidelines for conducting process evaluations of complex interventions [19]. A complex intervention is defined as an intervention with several interacting components [20], and the MRC outlines three essential components for evaluating complex interventions: implementation, mechanisms of impact, and context. Implementation can refer to how an intervention will be delivered within routine clinical practice, having shown efficacy in an outcome evaluation. However, this paper is concerned with another aspect of implementation: the extent to which the delivery of an intervention is achieved within the context of an RCT and the structures and processes through which an intervention is delivered as intended (ie, fidelity) [19]. For complex interventions such as DHIs, an important component of implementation fidelity is the degree to which participants engage with the intervention and use it as intended. Effective engagement requires participants to register with the program and then continue to use it and apply the recommended behavioral techniques over time. The nonuse of DHIs is a well-recognized challenge (eg, the Eysenbach Law of Attrition [21]) and can be considered in two parts: initial uptake (eg, registration and onboarding) and ongoing engagement.

To evaluate intervention implementation, the MRC guidelines for process evaluations suggest researchers assess (1) reach—the extent to which a target audience comes into contact with the intervention; (2) dose—how much of the intervention was delivered and received; (3) fidelity—the quality of what was delivered; and (4) adaptations—any modifications made to an intervention to achieve better contextual fit. The intended target audience for ORBIT was CYP with tic disorders; however, pertinent questions could be asked, such as whether there were socioeconomic biases in who was reached. In terms of dosage, the ORBIT protocol [15] states that the intervention should consist of 10 individual intervention chapters following a suggested frequency and a total duration of 10-12 weeks. The first four chapters delivered core content, including learning about tics and practicing suppressing tics. Completing these four chapters was designated as the minimum *dose* required for treatment completion by the ORBIT clinical team. There were six additional chapters offering reinforcement, further practice, and relapse prevention. For DHIs, the fidelity of delivery of the intervention is ensured by the web-based delivery platform. However, the intervention experienced by the user is highly dependent on the extent to which they engage with the intervention and use it as intended and the quality of delivery [22]. Hence, in this paper, we look at usage and the proportion of participants receiving the predefined *minimum effective dose* of four or more chapters. Finally, understanding adaptations to the intended intervention involves exploring whether they improve the contextual fit or compromise the functioning of the intervention [23] or whether they represent innovation or intervention drift [24]. Participants were able to modify various intervention components, such as the *tic stopwatch*, which was used to self-time the length of tic control. This study aims to conduct the first part of a two-stage process evaluation of the ORBIT trial outlined in the study protocol [25]. Part 1 focuses on intervention implementation by exploring the fidelity of delivery experienced by participants using usage statistics, reach, and acceptability of the intervention. It also investigates contextual factors associated with the observed variation in uptake and usage by examining the components specified in the MRC guidelines [19]. Part 2 will focus on the impact mechanisms, and the findings will be reported in a future article. Table 1 shows the two parts of the process evaluation: areas of research, explanatory data, and outcomes.

**Table 1:** Process evaluation parts, areas of research, explanatory data, and outcomes.

Research questions	Process evaluation components	Explanatory data	Outcomes
Part 1. Intervention implementation (What is implemented and how?)	Fidelity of implementation Dose of intervention delivered Adaptations Reach	Therapist contact (N=112) Intervention engagement (N=112) Usage metrics (N=112) Clinician (n=6), children and parent (n=20), and therapist (n=4) interviews	Engagement and satisfaction with the intervention
Part 2. Mechanisms of impact (How does it produce change*?)*	Mediators and moderators Unexpected pathways and consequences	Usage metrics Therapist contacts Clinician, children and parent, and therapist interviews	Relationship between engagement with the intervention and change in tic severity
Part 1 and 2. Context (How do factors external to the intervention affect intervention implementation and change?)	Factors related to fidelity of delivery (part 1) and improvement in tics (part 2)	Demographic data Clinician, children and parent, and therapist interviews Comorbidities Baseline severity of tics	Engagement with the intervention (part 1) Change in tic severity (part 2)

## Methods

### Study Design

This study followed the MRC guidelines [19] for the process evaluation of complex interventions. It used a mixed methods, longitudinal design to explore the implementation fidelity of a web-based intervention for CYP with tics [15] and the contextual factors that influenced the level of engagement.

### Participants

The sample included in the quantitative phase of the process evaluation consisted of key information from all participants (N=112) from the intervention arm of the RCT. The sample included in the qualitative component of the process evaluation consisted of interviews with children and parent participants (target n≥20), interviews with all therapists delivering the intervention or supervising the therapists, and interviews with referring clinicians (target n>5).

### Quantitative Data Collection

Quantitative process data were collected simultaneously with enrollment, intervention delivery, and outcome data collection in the main RCT.

#### Demographic and Clinical Data

Demographic and clinical information were recorded using a baseline demographic questionnaire. These data included the child’s age, residence (full postcode), gender, ethnicity, parental education level and occupation, all current suspected or confirmed diagnoses and interventions, and medication use.

#### Index of Multiple Deprivation

The index of multiple deprivation (IMD; 2019) is a relative measure of deprivation across seven different domains: income deprivation; employment deprivation; education, skills, and training deprivation; health deprivation and disability; crime; barriers to housing and services; and living environment deprivation [26]. On the basis of the 6-digit zip code, the rank of deprivation associated with participants’ area of residence was calculated from 32,844 small areas or neighborhoods in England, with higher ranks indicating greater deprivation. Ranks were recoded into quintiles, with 1 being the most deprived and 5 being the least deprived.

#### Yale Global Tic Severity Scale

The primary outcome measure used in the ORBIT intervention was the Total Tic Severity Score (TTSS), as measured by the Yale Global Tic Severity Scale (YGTSS). The YGTSS is a valid and reliable clinician-rated scale [27], which scores the severity of motor and vocal tics separately by evaluating the number, frequency, intensity, complexity, and interference of tics. Each domain was scored on a 0-5 scale, and 2 tic severity scores were given: total motor (0-25) score and total vocal (0-25) score, which when combined give the TTSS (0-50).

#### Mood and Feelings Questionnaire

The Mood and Feelings Questionnaire (MFQ) [28] is a 33‐item measure evaluating depressive symptoms rated on a 3‐point scale: 0 is *not true*, 1 is *sometimes*, and 2 is *true*. Total scores range from 0 to 66, with higher scores reflecting more severe depression. A cutoff score of ≥29 is generally used to suggest clinically significant depression [29].

#### Usage Metrics

Web usage data were collected and recorded from the participants throughout the trial. This included the following measures: number of chapters completed per child and per parent, total therapists’ time per child and per parent, individual therapist’s telephone time with participants, the volume of written communication (total number of characters) submitted by child and parent via the web-based system, total number of log-ins for child and parent, average time between each log-in (in days) for child and parent, and average pages visited per log-in for child and parent.

#### Satisfaction and Treatment Credibility

At the 3-week postrandomization point of treatment, all participants were asked to rate treatment credibility; two questions were asked: one relating to how well suited the participant felt the intervention was for helping CYP to manage their tics and the other question was about how much better they expected to feel as a result of the intervention. The responses were rated on a Likert scale of 0 to 4 for each question, with higher scores indicating higher treatment credibility. At the primary endpoint, all participants were asked to rate their satisfaction with the intervention. In total, eight satisfaction questions were asked with responses rated on a scale of 0-4, meaning that the overall satisfaction score was out of 32.

### Qualitative Data Collection

Interviews with therapists and therapist supervisors involved in the ORBIT trial were conducted early in the study and near the end of recruitment to gain an understanding of their experience at different time points. Interviews with referring clinicians were conducted at the end of recruitment. Interviews with children participants and one of their parents were conducted following completion of the intervention at the 3-month (primary endpoint) follow-up assessment in the main RCT to minimize the risk of bias in the outcomes. Recruitment for the interviews began in August 2018 and ended in October 2019.

All interviews were conducted face-to-face, via telephone, or via videoconference (WebEx or Skype). Younger children were interviewed together with their parents, whereas older children (eg, aged >13 years) were interviewed separately. Participants were purposively sampled so that a diverse range of views on the intervention were voiced [30]. This included ensuring that perspectives were heard from participants with a range of ages, gender, ethnicity, and level of interaction with the intervention. The overall sample enabled diverse intervention views and ensured that the data reached the saturation level [31]. In addition to the interviews, at the end of the treatment, all participants were asked to give their overall feedback on the intervention, to which they could provide open-ended responses. Table 2 demonstrates how various data sources contributed to the different components of implementation fidelity.

**Table 2:** Implementation fidelity components and data sources.

Data sources			Reach		Dose		Fidelity		Adaptations		Context
**Quantitative data sources**											
	Demographic and clinical data	✓^a^								✓	
	Usage metrics			✓							
	Treatment credibility and satisfaction					✓					
**Qualitative data sources**											
	Child interviews			✓		✓		✓		✓	
	Parent interviews			✓		✓				✓	
	Therapist and clinician interviews	✓				✓		✓		✓	
	End-of-treatment feedback questionnaire					✓					

**^a^**Associated data with implementation component.

### Data Analysis

Data were tested for normality using the Kolmogorov–Smirnov test. A principal component analysis was run to determine a composite measure of the level of engagement. Correlations between variables were examined using bivariate Spearman correlations, and a *t* test was performed to explore any significant differences between groups using chi-square tests to explore the differences between categorical variables. Multiple linear regression was used to identify predictors of engagement with the independent variables. All statistical analyses used a significance level of *P*<.05 and were conducted using SPSS Statistics 27 (IBM Corporation).

All interviews were recorded by using either videoconferencing software or a Dictaphone and were then transcribed verbatim. Transcripts were checked for accuracy against the recordings, with any corrections made as appropriate and anonymized for confidentiality purposes. As the process evaluation was a combination of exploration and description, the framework method [32] of analysis was used to identify, analyze, and report patterns within the transcribed interviews. Moreover, the steps outlined by Gale et al [33] were systematically followed to create an overall framework matrix using categories of engagement and potential moderators. Consistency of analysis was ensured through the use of a codebook and frequent meetings between researchers. Researcher bias was minimized through regular cross-checking of data and outcomes by the members of the research team.

The NVivo 12 software package (QSR International) was used to analyze the interview data. In addition, the end-of-treatment feedback questionnaire was exported to a Microsoft Excel spreadsheet, and quantitative content analysis [34] was performed. Overall, the findings from the qualitative analysis were linked to relevant quantitative engagement outcomes and contextual factors to assess which potential moderators may have influenced implementation fidelity and in what way, in an approach termed *triangulation* [35].

### Availability of Data and Materials

Some of the data generated or analyzed during this study are included in this paper and its supplementary information files or are available from the corresponding author upon reasonable request. The full data sets generated or analyzed during this study are also available from the corresponding author upon reasonable request.

### Ethical Considerations

Ethical approval for the study was obtained from the North West Greater Manchester Central Research Ethics Committee (REC: 18/NW/0079). We sought written parental consent and written informed assent or consent to participate in the study from CYP. This covered the process evaluation measures. All participants provided verbal consent to be audio-recorded for all interviews.

## Results

### Overview of Qualitative Sample

Semistructured interviews were conducted with children (n=20), parents (n=20), therapists (n=4), and clinicians (n=6). The average age of the child interviewees was 12 years (SD 2.1); range 9-16 years), of which 80% (16/20) were male and 20% (4/20) were female. Most participants were White (18/20, 90%). The mean TTSS was 28.8 out of 50 (SD 7.2), with a range of 13-45 for the child interviewees. All 20 interviews with the parents were with the CYP’s mothers, with all 20 having completed at least some further education. One of the therapist interviewees was a therapist’s supervisor, and half of the clinicians (3/6, 50%) were consultant psychiatrists.

### Reach

Participants were eligible for the study if they were aged between 9 and 17 years and competent to provide written, informed consent (parental consent for a child aged <16 years), had a suspected or confirmed tic disorder (as confirmed by scores on the YGTSS), and had broadband internet access and regular use of a computer, with mobile phone text messaging facilities. Patients were excluded from the study if they had received any form of structured behavioral intervention for tics within the preceding 12 months, had a change of medication for tics within the previous 2 months, had any diagnoses of alcohol or substance dependence, psychosis, suicidality, anorexia nervosa, or moderate or severe intellectual disability, were an immediate risk to self or others, and/or parents or children were not able to speak, read, or write English.

A total of 445 families expressed an interest in participating in the study either through self-referral via the *Tourettes Action* charity website (n=251) or clinic referral (n=194); however, 47 were subsequently uncontactable, and 90 were ineligible to participate. Of the 308 potentially eligible CYP, 84 (27.3%) families declined to participate, and 50% (112/224) of CYP (90 males and 22 females) with a mean age of 12.2 (SD 2) years were randomized to the intervention arm of the ORBIT trial and included in the process evaluation. The sample was predominantly White (96/112, 85.7%) and well-educated, with more than half (60/112, 53.5%) of the participants’ mothers having completed university or higher education.

The median IMD rank was 19,318 and ranged from 147 to 32,668 (out of 32,844). Of the 112 participants, 8 (7.1%) were in the most deprived quintile 1, 31 (27.7%) in quintile 2, 18 (16.1%) in quintile 3, 26 (23.3%) in quintile 4, and 29 (25.6%) in the least deprived quintile 5. Although the reach of the intervention was not limited geographically, for research purposes, participants had to attend a baseline screening assessment at either the Nottingham study site (57/112, 50.9%) or the London study site (55/112, 49.1%) depending on personal preference and/or location of residence. All participants were based in England, with 56.3% (63/112) of participants living in towns, 26.7% (30/112) living in cities, and 16.9% (19/112) living in villages.

In terms of clinical characteristics, the intervention reached a moderately severe symptomatic sample with a mean TTSS of 28.4 out of 50 (SD 7.7) ranging from 12 to 50. Most participants (98/112, 87.5%) were not on any medication for their tics, and less than half of the overall intervention sample had no diagnosed or suspected comorbidities (51/112, 45.5%). Among those who had a comorbid diagnosis, the most common was anxiety disorder (34/112, 30.4%), followed by attention-deficit/hyperactivity disorder (26/112, 23.2%). An assessment of depressive symptoms by the MFQ showed a mean score of 16.3 out of 66 (SD 11.3), with 12.5% (14/112) of participants scoring above the cut-off (≥29), suggesting clinically significant depression [29] (Table 3).

It was not possible to interview people who had not taken part in the study, so the qualitative data threw little light on reach. However, a clinician identified that some families were worried about the level of commitment involved and associated travel to one of the study sites (quote 1) under the theme *clinician perceptions of and contribution to recruitment* (see Multimedia Appendix 1 for a full list of framework categories and themes and Multimedia Appendix 2 for a full list of quotes). Another clinician highlighted the lack of access to children with intellectual disabilities (quote 2). Finally, one of the clinicians struggled to gain her colleagues’ interest in the intervention despite numerous attempts (quote 3).

**Table 3:** Demographic and clinical characteristics of participants in the Online Remote Behavioral Intervention for Tics trial intervention group (N=112).

Variable			Intervention group
**Gender, n (%)**			
	Male	90 (80.4)	
	Female	22 (19.6)	
**Study site, n (%)**			
	Nottingham	57 (50.9)	
	London	55 (49.1)	
**Ethnicity, n (%)**			
	White	96 (85.7)	
	Asian	7 (6.2)	
	Mixed race	3 (2.7)	
	Other	6 (5.4)	
**Supporter, n (%)**			
	Mother	93 (83)	
	Father	16 (14.3)	
	Other	3 (2.7)	
**Highest level of education (mother), n (%)**			
	Did not complete compulsory education	3 (2.7)	
	Completed compulsory secondary education	16 (14.3)	
	Completed further education	33 (29.5)	
	Completed university or higher education	43 (38.4)	
	Completed postgraduate taught degree	11 (9.7)	
	Completed doctorate or medical degree	6 (5.4)	
**Highest level of education (father), n (%)**			
	Did not complete compulsory education	2 (1.8)	
	Completed compulsory secondary education	29 (25.9)	
	Completed further education	35 (31.2)	
	Completed university or higher education	29 (25.9)	
	Completed postgraduate taught degree	10 (8.9)	
	Completed doctorate or medical degree	7 (6.3)	
**Method of referral, n (%)**			
	Self	69 (61.6)	
	Clinic	43 (38.4)	
Age (years), mean (SD)			12.2 (2)
IMD^a^ rank, median (range)			19318 (147-32,668)
No tic medication, n (%)			98 (87.5)
On tic medication, n (%)			14 (12.5)
Comorbidities, n (%)			61 (54.5)
No comorbidities, n (%)			51 (45.5)
TTSS^b^ baseline score, mean (SD)			28.4 (7.7)
MFQ^c^, mean (SD)			16.3 (11.3)

^a^IMD: index of multiple deprivation.

^b^TTSS: Total Tic Severity Score.

^c^MFQ: Mood and Feelings Questionnaire.

### Dose

Child participants completed an average of 7.5 (SD 2.7; Table 4) chapters, and their parents completed an average of 7.6 (SD 2.8; Table 5) out of 10 chapters of the intervention, indicating high engagement. Only 11.6% (13/112) of child participants and 15.2% (17/112) of parents failed to meet the criteria for treatment completion (ie, the minimum of the first four chapters completed as per protocol) with a total of 88.4% (99/112) of child participants and 84.8% (95/112) of parents completing their treatment, meaning that adherence to the intervention was high. Indeed, 41% (46/112) of CYP and 46.4% (52/112) of parents completed all 10 intervention chapters, and only 1 child participant failed to complete any chapters. Participants were given 10 weeks of supported therapeutic input to complete their treatment chapters. In some circumstances, such as holidays or particularly busy periods, 1 or 2 weeks were added to supplement this time. Although most families (73/112, 65.2%) completed their therapy within 10 weeks, 34.8% (39/112) required extra time to complete treatment. Child participants logged onto the web-based treatment platform an average of 19.8 (SD 10.9) times throughout the 10-12 weeks, with an average of 4.2 (SD 2.6) days between log-ins. In terms of total interactions with their assigned therapist, child participants required their therapist’s assistance on the web for an average of 59 minutes and 14 seconds (SD 29 min and 08 s) throughout treatment, resulting in approximately 6 minutes per child each week. In contrast, parents interacted on the web with their assigned therapist for an average of 1 hour, 23 minutes, and 55 seconds (SD 42 min and 45 s), resulting in approximately 8 minutes per parent each week. Of the 112 CYP, only 2 (1.8%) were contacted by telephone by their assigned therapist, whereas of the 112 parents, 49 (43.7%) were contacted by telephone by their assigned therapist.

Interview data relating to participants’ *perceptions of the ORBIT organization* covered the implementation component of the dose. Although most participants felt that the intervention was just the right length, some CYP wished to have a longer time to access their therapist (quote 4). A child felt that the intervention could have been condensed to make it shorter (quote 5). On the whole, parents agreed with their child that the dose received was just right, with a parent claiming that if it were longer, it would have negatively affected engagement levels (quote 6).

**Table 4:** Usage data for child participants in the Online Remote Behavioral Intervention for Tics trial intervention group (N=112).

Variable	Value	
	Median (range)	Mean (SD)
Chapters completed	8 (0-10)	7.5 (2.7)
Total therapist time (hh:mm:ss)	00:53:57 (00:07:27-03:11:08)	00:59:14 (00:29:08)
Telephone time with therapist (hh:mm:ss)	00:00:00 (00:00:00-00:18:44)	00:00:10 (00:01:46)
Number of log-ins	19 (3-57)	19.8 (10.9)
Number of days between log-ins	3 (1-16)	4.2 (2.6)
Number of pages visited per log-in	15 (7-38)	16.9 (5.8)
Total number of characters submitted	2507 (238-8749)	2784 (1608)

**Table 5:** Usage data for parents in the Online Remote Behavioral Intervention for Tics trial intervention group (N=112).

Variable	Value	
	Median (range)	Mean (SD)
Chapters completed	9 (1-10)	7.6 (2.8)
Total therapist time (hh:mm:ss)	01:15:33 (00:22:01-04:48:19)	01:23:55 (00:42:45)
Telephone time with therapist (hh:mm:ss)	00:00:00 (00:00:00-00:49:00)	00:04:06 (00:07:41)
Number of log-ins	18 (3-50)	20.4 (11.4)
Number of days between log-ins	4 (0-19)	4.2 (2.7)
Number of pages visited per log-in	17 (9-36)	17.4 (5.2)
Total number of characters submitted	6533 (346-29,631)	7286 (5093)

### Fidelity

At the 3-week point of postrandomization, participants were asked to rate treatment credibility. Treatment credibility was rated highly by the child participants, with a mean score of 6.4 out of 8 (SD 1.5). Furthermore, at the primary endpoint, participants were asked to rate their overall satisfaction with the intervention. Child participants were highly satisfied with the intervention, with a mean score of 24.8 out of 32 (SD 5.2). At the end of the treatment, participants were asked to give their feedback on the intervention within the web-based platform, and they were able to give open-ended responses. Only 59.8% (67/112) of child participants provided feedback. From the conducted quantitative content analysis, four categories emerged relating to implementation fidelity, namely *limitations of ORBIT* (51/67, 76%), which captured how participants felt that overall ORBIT was helpful however was limited by certain factors; *ORBIT as a suitable treatment* (49/67, 73%), which suggested that participants felt that the web-based delivery of treatment for tic disorders was suitable; *problems with using ORBIT* (20/67, 30%), which captured those participants who stated that they felt ORBIT was not helpful to them or was associated with negative factors; and *feeling supported* (19/67, 28%; see Multimedia Appendix 3 for a full list of content analysis categories and codes), where participants mentioned that they felt supported in a way they had never been before (eg, by their therapist). The main code relating to *limitations of ORBIT* centered on *improvement required* (n=33). This code captured anything related to the intervention being unhelpful or inappropriate. Examples include repetitiveness of treatment, the treatment being too short or too long, unhelpful aspects, and suggested improvements. Two child participants reported technical issues with the ORBIT platform, which was related to intermittent problems with connectivity. Despite this, many participants felt the intervention was acceptable as a treatment, with the largest number of participants being coded as *a positive experience of ORBIT* (n=42), which was part of the category *ORBIT as a suitable treatment* and related to being pleased by having taken part and finding it enjoyable while recommending the treatment to other CYP with tic disorders.

Although satisfaction was rated highly, some participants felt that the role of the therapist was somewhat misleading. This was captured by the theme *expectations of role of the therapist*. Some felt that a therapist was not needed for the delivery of the intervention (quote 7). Some parents agreed with the sentiment that they could have completed the therapy without the assistance of a therapist (quote 8).

The term *therapist* itself was felt to be somewhat misjudged as a label, and they viewed the therapist more as a motivator (quote 9). The therapists themselves concurred with this, and perhaps they should not have been called a *therapist* within the ORBIT study (quote 10).

At the end of the interviews, participants were asked if they had any recommendations to improve the intervention, and the overriding majority felt that a mobile application was needed in future iterations of the intervention (quote 11). Some of the older CYP felt that the content and presentation of the intervention were childlike and aimed more toward younger participants. Therefore, they felt there could be two separate versions: one for teenagers and one for young children (quote 12).

### Adaptations

Regarding adaptations, the intervention did not appear to evolve in any way from the original plans. Instead, there appeared to be consistency in the way the intervention was delivered and received. Interviews with therapists confirmed that consistency was maintained during delivery. For example, they created a list of standardized responses to common queries (quote 13). Parts of the intervention were designed to be adapted by the user and tailored to their needs and preferences, such as the *tic stopwatch* and *tic ladder* (hierarchy of exposure exercises), which was confirmed in the interviews (quote 14). Another participant adapted the intervention to make it easier to complete by altering some activities to make them more user friendly (quote 15).

### Contextual Factors Influencing Intervention Implementation

To establish a measure of intervention implementation that captured both the breadth and depth of participants’ usage, a principal component analysis with varimax (orthogonal) rotation was conducted on the seven items related to the dose of intervention received. The analysis suggested a two-factor model. The strongest factor accounted for 47% of the variance (Eigenvalue 3.3; Table 6) and seemed to capture the strength of engagement with the intervention. Factor scores ranged from −2.65 to 2.26 with a mean of 0.001 (SD 0.99), and these scores were used as the engagement measure.

A two-tailed *t* test found that participants enrolled at the London site (mean 0.25, SD 0.90) scored significantly higher on engagement than those enrolled at the Nottingham site (mean −0.22, SD 1.03; t_109_=−2.58; *P*=.01). Moreover, those who were self-referred (mean 0.16, SD 0.94) scored higher on engagement than those referred by clinics (mean −0.24, SD 1.04; t_109_=−2.06; *P*=.04). Spearman rho (ρ) correlations were used to determine the association between engagement and various contextual factors. CYP’s engagement factor scores were strongly correlated with parents’ chapter completion (n=111; ρ=0.73; *P*<.001) and moderately correlated with therapist time for parents (n=111; ρ=0.46; *P*<.001). There were no significant relationships between the CYP’s engagement factor score and age, parental education, IMD, TTSS at baseline, or MFQ baseline score. There were also no statistically significant relationships among the child’s gender, comorbidities, or use of tic medication and CYP’s engagement.

Multiple linear regression was conducted, with CYP’s engagement factor score as the dependent variable, and site, child’s age, child’s gender, IMD, TTSS, referral method, parental education, therapist time for parents, and parents’ chapter completion as the independent variables. The results of the simultaneous regression indicated that, collectively, the independent variables had a significant amount of variance in the CYP’s engagement factor score (*F*_10,100_=20.84; *P*<.001; *R^2^*=0.64). There was no evidence of multicollinearity, with all tolerances above 50% and all variance inflation factors below 2. Only parents’ chapter completion (β=.69; t_110_=10.18; *P*<.001) and therapist time for parent (β=.19; t_110_=2.95; *P*=.004) were significant independent predictors in the model.

The theme of *parental persuasiveness* was generated in the framework category *participant contextual factors*. Many of the parents interviewed outlined that they were often the main motivating force behind their child’s level of engagement by reminding their child to practice the learned techniques (quote 16). Some parents found motivating their child to engage very challenging (quote 17), and this was even more challenging for those with children who have comorbidities (quote 18).

Some parents found it difficult to support their children because of hectic schedules, which was captured by the theme of *busy lives* (quote 19). Although under the theme of *high motivation levels*, we found highly engaged CYP without their parents’ persuasion (quote 20).

**Table 6:** Summary of principal component analysis for children’s usage data for the Online Remote Behavioral Intervention for Tics intervention (n=111).

Item	Factor loadings	
	Factor 1: engagement	Factor 2: sporadic use
Number of log-ins	0.90	—^a^
Chapters completed	0.79	—
Total therapist time per child	0.76	—
Total number of characters submitted	0.74	—
Number of days between log-ins	−0.63	0.54
Number of pages visited per log-in	−0.41	0.80
Telephone time with the therapist	−0.44	−0.46
Eigenvalue	3.3	1.5
Percentage of variance	47	21

^a^Item not loaded onto factor.

## Discussion

### Principal Findings

This process evaluation used a mixed methods approach to investigate the extent to which the ORBIT intervention was implemented as planned within the RCT and explore participants’ experiences with the intervention and the contextual factors influencing children’s engagement. Doing so made it possible to identify reasons for variation in uptake, usage, and engagement, reflect on how implementation may ultimately give us greater confidence in the outcomes, and outline lessons for potential future implementation within routine care. The uptake of the intervention was high, with 88.4% (99/112) of participants receiving the predefined minimum effective dose of the first four chapters completed. The median uptake was eight chapters, and only 1 child failed to access any chapters. Fidelity of delivery was also excellent, with participants reporting high levels of satisfaction and acceptability.

The intended sample of CYP with a diagnosed tic disorder was reached, with 7.1% (8/112) of families residing in the most deprived areas (IMD quintile 1) and more than a quarter (29/112, 25.6%) of the families residing in the least deprived areas (IMD quintile 5). As more than half (60/112, 53.5%) of the CYP’s mothers had completed university education, against a UK average of 42% [36], it seems that more advantaged families may have been overrepresented. Perhaps the requirement to have broadband internet access and regular use of a computer with mobile phone text messaging facilities to participate in the study may have differentially impacted participants in the most deprived IMD quintile. This is a concern, as one of the aims of the ORBIT was to increase access to evidence-based therapeutic interventions for CYP with tic disorders. In particular, access to services is generally limited to those from lower economic backgrounds [37]. However, the initial baseline visit with associated travel may have been a disincentive to more disadvantaged families—a limitation that would not be relevant if ORBIT was delivered entirely remotely in routine care rather than as part of an RCT. Moreover, there is no evidence that socioeconomic factors influence CYP’s engagement with ORBIT. Furthermore, children’s age, tics severity, well-being, and comorbidities did not appear to influence the child’s level of engagement with the intervention, providing further evidence that it would have a wide reach within routine clinical care. However, because of the various factors related to this RCT as opposed to routine care, caution should be taken when interpreting the results of this study concerning reach.

The London study site, self-referral, and higher parental engagement were all associated with higher levels of engagement. The London site is a world-renowned center of excellence for pediatric care, which may have increased parents’ motivation for treatment. However, the only independent predictor of child engagement in the multivariate analysis was the level of parental engagement with intervention, as measured by their chapter completion and time with the therapist. This is consistent with previous literature [38-41], which found that parental involvement was particularly key for younger CYP to assist with their engagement with therapeutic interventions, which in turn leads to better outcomes [42-44]. It has been shown in the literature that parental engagement may impact a provider’s ability to implement parent- and family-focused evidence-based treatment with fidelity [42]. Therefore, it is crucial to understand the role of parental support in the implementation of DHIs for children, as without attention to the key processes of child and family engagement, efforts to improve the effectiveness and efficiency of the treatment are less likely to succeed. Furthermore, it is crucial to assess whether parental support also predicts intervention efficacy and the mechanisms through which its impact is achieved.

An interesting finding was the use and interactions with the therapists in this study. Therapists interacted on the web with their assigned child participants an average of 6 minutes per child each week, which is lower than the 24 minutes average time per week participants interacted with their therapist in the Swedish pilot trial, on which ORBIT is based [14]. However, in the United Kingdom, study therapists were encouraged to use preprepared scripts to respond to the participants. Their responsibilities involved reinforcing the ORBIT treatment material with the aim of spending around 6 minutes a week in response to each child. Detailed analysis of the content of therapists’ interactions is outside the remit of this study, but it is apparent from qualitative interviews that many participants felt that the term *therapist* was somewhat misleading. Some participants felt that *therapist* had connotations of a clinically-trained individual delivering an intervention. This may have limited their reliance on therapists. Therefore, in any implementation of this intervention within routine health care, it would be sensible to alter the title to *coach*, *guide*, or *mentor* as this better reflects the therapist’s role.

### Strengths and Limitations

To the best of our knowledge, this study is one of the first to conduct an in-depth mixed methods process evaluation of a complex intervention aimed at CYP with Tourette syndrome and chronic tic disorders. A number of important findings emerged from the process data, which helped us characterize the intervention’s implementation within an RCT and provided lessons for potential future implementation within routine care. However, these lessons can only be fully realized once the main RCT outcome data have been analyzed. Furthermore, a principal component analysis of participants’ usage data provided an objective, reliable, and comprehensive measure of engagement to explore the role of contextual factors.

However, this study has some limitations. First, there was the issue of potential recruitment bias. It may have been that the more motivated families self-referred to the trial and that recruitment from clinics was skewed toward punctual, frequent attenders, in contrast to patients with multiple missed appointments. This may have limited the power of this process evaluation to detect socioeconomic biases in engagement. Second, although comprehensive, the information on uptake cannot fully capture the quality and quantity of engagement with the ORBIT. For example, indices such as chapter completion, number of pages visited, and number of log-ins may not fully capture factors such as level of attention to practice exercises. Finally, and perhaps most crucially, a major limitation was that it was not possible to interview those who had not taken part in the RCT or reach those who had withdrawn early from the study. Their perspective is vital to fully understand the factors influencing engagement with DHIs.

### Conclusions

We conclude that the intervention had high fidelity of delivery and was evaluated positively by CYP, although some participants suggested some minor improvements, and the nature of the RCT may have constrained reach. Parental engagement was a strong, independent predictor of intervention implementation, which has important implications for designing and implementing digital therapeutic interventions in child and adolescent mental health services.

## Appendix

Multimedia Appendix 1 Analytic framework.

Multimedia Appendix 2 Qualitative quotes.

Multimedia Appendix 3 Content analysis tables.

## Abbreviations

CYP: children and young people
DHI: digital health intervention
IMD: index of multiple deprivation
MFQ: Mood and Feelings Questionnaire
MRC: Medical Research Council
NHS: National Health Service
NIHR: National Institute for Health Research
ORBIT: Online Remote Behavioral Intervention for Tics
RCT: randomized controlled trial
TTSS: Total Tic Severity Score
YGTSS: Yale Global Tic Severity Scale
