# Supplementary material for: Fidelity of Delivery and Contextual Factors Influencing Children’s Level of Engagement: Process Evaluation of the Online Remote Behavioral Intervention for Tics Trial
Source: J Med Internet Res. 2021 Jun 21;23(6):e25470. doi: 10.2196/25470 (PMC8277316; doi:10.2196/25470)
Supplement: Multimedia Appendix 1 [file jmir_v23i6e25470_app1.docx]

| **CATEGORY 1** | **DESCRIPTION** |
| --- | --- |
| **Motivation for participating** | |
| To remove tics | *Participants stated that they wanted to participate so that their or their child’s tics will be gone completely or decrease in severity and frequency* |
| To help others/research | *Altruistic reasons for participating* |
| Some sort of support | *Lack of support from services therefore looking for any type of support available* |
| Hoped to learn more about tics | *Lack of information and knowledge of tics so wanted to learn more to help themselves/their child* |
| Due to it being done online | *Participants motivating factor was because it was online* |
| **CATEGORY 2** |  |
| **Initial response to ORBIT** | |
| Participant responsiveness | *How participants and therapists initially responded to ORBIT. Includes assessments by participants about the outcomes and relevance of ORBIT* |
| Quality of ORBIT trial description | *Degree to which the ORBIT trial was sufficiently and clearly described* |
| Quality of delivery | *Concerns whether the intervention was delivered in a way appropriate to achieving what was intended including participants thoughts on therapists* |
| Strategies to support therapists | *Refers to strategies such as provision of manuals, guidelines, training, and supervision* |
| Clinician perceptions of and contribution to recruitment | *Refers to consistency of recruitment procedures, perceptions of reasons for non-participation among potential participants, and subgroups less likely to participate* |
| Perception of initial recruitment strategies | *Includes participants views on the initial telephone screening and baseline assessment* |
| Relevance of questionnaires | *Participants views on the relevance of the questionnaires to themselves* |
| Expectations of role of the therapist | *Perception that ‘therapist’ was a misleading name* |
| **CATEGORY 3** |  |
| **ORBIT program content** | |
| Perceptions of ORBIT organisation | *Includes views on whether ORBIT was an appropriate length, the structure of sessions, and frequency of therapist contact* |
| Lack of fit between content and child | *Includes judgment on the videos, animations, appropriateness to child’s age, and missions* |
| Useful and enjoyable program resources | *What participants felt they have learnt from ORBIT (e.g. strategies parents have made as a result) and what were the most useful and enjoyable resources used* |
| Ease of use | *The ability to use ORBIT even if you lack IT skills* |
| ORBIT recommendations | *What participants and therapists feel could be added or removed in ORBIT to improve the program* |
| **CATEGORY 4** |  |
| **Mechanisms of impact** | |
| Features of online therapy to support tic reduction | *Perception of online therapy working to help reduce tics and related behaviours including acceptability and satisfaction with ORBIT* |
| Perceived benefits of therapist support | *Having a therapist provided continued focus and motivation and the ability to answer any queries* |
| Limitations of online therapy | *ORBIT was limited by being delivered online and participants would have preferred face-to-face therapy* |
| Working together | *Instances of parent and child going through ORBIT together and the impact on how ORBIT was used and their relationship* |
| Unanticipated consequences | *This captures anything that happened unexpectedly as a result of ORBIT* |
| **CATEGORY 5** |  |
| **Intervention outcomes** | |
| Level of control | *The child has better control over their tics in their daily life* |
| Expectations vs. reality | *Considering the participants expectations of ORBIT, what has the reality been in outcomes* |
| Long-term outcomes | *Going forward what does the future hold for participants. This includes anything the participant has said about future plans regarding use of services and whether they will continue to use ORBIT* |
| Routine clinical practice | *This refers to what clinicians feel are the main enablers or barriers to implementation of ORBIT in clinical practice (e.g. lack of funding)* |
| Improved self-esteem and confidence | *The intervention improved the child’s sense of self, confidence, and quality of life* |
| Improvement in tics | *The intervention improved the frequency and severity of the child’s tics* |
| Impact on parent | *The intervention had a positive impact on the parent in caring for their child* |
| **CATEGORY 6** |  |
| **Intervention characteristics that enabled implementation** | |
| Flexibility of online therapy | *Being able to do online therapy at your own time and pace is seen as a positive* |
| Therapist support | *Having therapist support was seen as essential* |
| Use of computers | *Children and young people prefer using computers over face-to-face contact* |
| Perceived impact of therapy | *If the participant started to perceive the intervention as having a positive impact they were more likely to engage* |
| Adaptations | *Participants tailoring the intervention by making modifications to suit their needs* |
| **CATEGORY 7** |  |
| **Trial related enablers to implementation** | |
| Opportunity to discuss tics | *This captures how children could open up and talk about their tics to someone other than their family members for the first time* |
| Follow-ups | *Having continuous support through follow-up appointments had a positive impact* |
| Financial reimbursement | *The use of vouchers as a reward for completing each follow-up aided implementation as well as expenses being reimbursed for initial baseline assessment* |
| Trust in experts | *Refers to how participants felt positive about the therapy as it was conceptualised and delivered by tic experts* |
| **CATEGORY 8** |  |
| **Trial related barriers to implementation** | |
| Staffing resources | *Staffing issues and demands placed on the ORBIT team affected quality of implementation* |
| Demand on participants | *Trial related demands on participants (e.g. travelling long distances for baseline assessment and ability to participate in follow ups)* |
| Therapists workload | *Overworking of therapists affected quality of implementation* |
| Therapeutic relationship | *Therapists struggled to build an alliance with participants due to lack of visibility* |
| Therapists background and confidence | *Therapists didn’t feel confident in their qualifications or ability to carry out their role expertly* |
| **CATEGORY 9** |  |
| **Intervention characteristics that supported tic reduction** | |
| Visualisation of progress | *Participants were more likely to engage better as they could see the progress they were making and competing to beat their times on tasks* |
| Use of rewards | *Children were more likely to engage as they knew they would be receiving a reward for completing the tasks and practises* |
| **CATEGORY 10** |  |
| **Intervention characteristics that hindered engagement** | |
| Repetitiveness | *Participants found the content highly repetitive and therefore would lose motivation and disengage* |
| Perceived lack of utility | *Participants would disengage if they didn’t see an immediate impact on their tics* |
| Lack of interaction | *Participants wanted more face-to-face contact with therapists even if this was via videoconferencing and were more likely to stop persisting with ORBIT if they didn’t receive this* |
| Negative impact on tics | *As can be an effect of ERP, tics began to worsen and therefore participants would disengage* |
| Lack of relevance | *If participants began to feel some of the components of the intervention were not relevant to them, they would disengage* |
| Perceptions of lack of engagement | *Reasons clinicians and therapists felt that some may have not engaged as well as others* |
| **CATEGORY 11** |  |
| **Participant contextual factors** | |
| Perceived utility | *Participants who expected long term benefit of ORBIT persisted to complete the chapters and tasks* |
| High motivation levels | *Participants who were highly motivated to engage in ORBIT continued to engage with it* |
| Parental persuasiveness | *Parents were the main motivating force behind their child’s level of engagement* |
| **CATEGORY 12** |  |
| **Family contextual factors** | |
| Life stressors | *This captures how families struggled with ORBIT due to various stressors (e.g. child about to move to a new school)* |
| Busy lives | *The context of how families fit ORBIT into their everyday lives despite being busy (e.g. work, extracurricular activities)* |
| Family dynamics | *Parents with other children who also have neurodevelopmental or health issues* |
| School life | *How the exam period or holidays affected participation in ORBIT* |
