# Supplementary material for: Fidelity of Delivery and Contextual Factors Influencing Children’s Level of Engagement: Process Evaluation of the Online Remote Behavioral Intervention for Tics Trial
Source: J Med Internet Res. 2021 Jun 21;23(6):e25470. doi: 10.2196/25470 (PMC8277316; doi:10.2196/25470)
Supplement: Multimedia Appendix 2 [file jmir_v23i6e25470_app2.docx]

## Qualitative quotes

**Quote 1**: “So children quite often with autism…other kind of family reasons where I think they were just worried about the level of that kind of commitment to…an intervention to be able to kind of travel to Nottingham or London for the initial assessment” (Clinician 3, Psychiatrist).

**Quote 2**: “So say for example they’ve got severe intellectual inabilities so they’re non-verbal you know so clearly they’re not gonna be able to access the trials and things. I mean even somebody with a mild…intellectual disability to be honest if it was on the low end of the mild so kind of like between 50 to 60 in the IQ kind of thing…you would struggle to, you know, to access it” (Clinician 1, Psychiatrist).

**Quote 3**: “So but the interesting thing is to get clinicians interested in it and thinking about the children because we have a big Trust with three areas and I have sent it out over and over and over and over again and I think the uptake has been really low from the other…professionals” (Clinician 2, Psychiatrist).

**Quote 4**: “I just liked doing the whole bit of ORBIT and chatting to my therapist but I think it was too short. Cause I could only chat to my therapist for 10 weeks, but then we had a full year logging on to ORBIT but we could not chat to our therapist which I found a bit annoying” (Child 20, 12 years old).

**Quote 5**: “9 weeks with 12 chapters. Make the chapters shorter. Some of them are like 13 pages like you have to do the questions. Like those pages questions” (Child 26, 9 years old).

**Quote 6**: “Just the right length. I think if it’d been any longer he’d have got he wouldn’t have engaged as much” (Parent 26, Mother).

**Quote 7**: “Like that just I don’t like emailing so I think I felt a bit awkward cause I didn’t really know how to write back but I felt most of the comments were quite generic…I don’t know just I’d say something and [therapist] be like ‘oh well done’…but I don’t think [therapist] necessarily has to be there. I think you could have done it on your own” (Child 21, 15 years old).

**Quote 8**: “I probably could have done without the therapist because I would want a therapist to advise me about [child’s name] tics I didn’t need advising about using the therapy, does that make sense?” (Parent 25, Mother).

**Quote 9**: “I don’t know that the therapist was of any use. We didn’t utilise the therapist I don’t think. It was more sort of it felt like they were cheering you on…they are more like a motivator than a therapist I think. I kind of maybe expected a little too much from the ORBIT study” (Parent 30, Mother).

**Quote 10**: “I think part of it would come down to whether we would want to use the word ‘therapist’ within ORBIT because there’s a lot of semantics and meaning about that word and I’m not sure off the top of my head if therapist or…what’s the lay meaning of therapist basically? Does that mean psychotherapist, does that mean someone who’s got a doctorate, who knows? So, everyone could…participants come into that with their own meaning and it also assumes that I…they’ve got expectations about what a therapist is, it assumes that I’m the expert and I really felt like I wasn’t in this. My supervisors were experts” (Therapist 1).

**Quote 11**: “I mainly focused on…wanting to beat my score and like I couldn’t actually put that on when I was like…I couldn’t actually put it online when I was…just like in lesson or when I was like doing it…watching TV, just like do the stop clock on my phone. So I think like if they had an app or something” (Child 27, 13 years old).

**Quote 12**: “The layout and stuff was very much directed to younger kids…and I think if there was like a separate part of ORBIT that was for more like teenagers and stuff…and…the videos were a bit more…accustomed to young children. And…I think if there was just a bit there that was more directed to teenagers I think it would be better in that way” (Child 14, 13 years old).

**Quote 13**: “We had standardized documents, of like a collection of standardized responses so any time we’d come across something unique or difficult or not immediately obvious to answer, after sort of emailing around and reviewing potential answers we’d obviously say how to come up with an answer to send to the participant and once I’d done so, I’d add a section into the collection of responses and add it in. So basically, we had something we could look at and call upon when we see someone and go ‘look, we’re not sure how to answer that, let me check this document’ and then you can see if there was anything similar, or it’s been answered before…that was very useful…” (Therapist 2).

**Quote 14**: “I had to answer questions in the chapters and when I finished it I could go back and change it and I could change my ladder when I do my tics and where I do my tics most often and my tic list of what I have. I liked the idea that I could change it. And it helped me” (Child 20, 12 years old).

**Quote 15**: “We…changed some of the activities that like…so one of them was like…doing trying to suppress your tics whilst focussing only on your tics. But I really wasn’t able to do that one at all really so we did that while I was watching TV or like being on my phone. So we changed some bits” (Child 22, 15 years old).

**Quote 16**: “If he’s got a really bad tic and I’ll say to him you know, [child’s name] use your tic timer in your head, try and see how long you can do he will then do it…but he doesn’t really use the techniques himself without being reminded to…So I suppose that was a little bit of a disappointment” (Parent 15, Mother).

**Quote 17**: “Obviously for me trying to keep [child’s name] engaged…on the computer and with the time aspect…you know that was the challenging part” (Parent 28, Mother)

**Quote 18**: “I knew I’d have to help motivate him…cause he has ADHD…he’s got easily distracted and…he hasn’t got a great attention span but that was fine because I knew the importance of it so I was fully aware when I went into it” (Parent 8, Mother).

**Quote 19**: “It was a challenge as I said because I work 4 days a week…ideally it would have been better to do it after school when we had plenty of time. It was a bit sort of frantic at times…you know trying to fit cooking tea in and…try and fit it in before bedtime so from that point of view…as I said I knew that would be our biggest challenge was the time aspect…so yeah it was a challenge” (Parent 28, Mother).

**Quote 20**: “[Child’s name] was fully engaged and I think the whole thing made him feel quite special. I think the fact it was targeted. The fact it was all about tics and it was educational and he was seeing other kids with it. It was all positive” (Parent 6, Mother).
