# Supplementary material for: Fidelity of Delivery and Contextual Factors Influencing Children’s Level of Engagement: Process Evaluation of the Online Remote Behavioral Intervention for Tics Trial
Source: J Med Internet Res. 2021 Jun 21;23(6):e25470. doi: 10.2196/25470 (PMC8277316; doi:10.2196/25470)
Supplement: Multimedia Appendix 3 [file jmir_v23i6e25470_app3.docx]

## Content analysis tables

| Number of participants in the ERP allocation | | | Number of participants who did not report any codes (did not complete Chapter 10 or send a message highlighting feedback) | | |
| --- | --- | --- | --- | --- | --- |
| Child | Supporter | Total | Child | Supporter | Total |
| 112 | 113 (One Child had two supporters) | 225 | 45 / 112 | 41 / 113 | 86 / 225 |

| Category | Number of participants reported a code related to this category. | | | Number of participants who reported a code related to this category more than once. | | |
| --- | --- | --- | --- | --- | --- | --- |
|  | Child | Supporter | Total | Child | Supporter | Total |
| A – Improved Experience | 63 | 68 | 131 | 59 | 62 | 121 |
| B – Feeling Supported | 19 | 41 | 60 | 2 | 21 | 23 |
| C – Limitations of ORBIT | 51 | 55 | 106 | 29 | 43 | 72 |
| D – ORBIT is Suitable | 49 | 59 | 108 | 34 | 48 | 82 |
| E – Problems associated with ORBIT | 20 | 39 | 59 | 3 | 24 | 27 |

| Code | Number of participants reported this code. | | | Number of participants who reported this code more than once. | | |
| --- | --- | --- | --- | --- | --- | --- |
|  | Child | Supporter | Total | Child | Supporter | Total |
| A - Increased tic control | 60 | 47 | 107 | 40 | 24 | 64 |
| A – Increased Knowledge/Awareness | 19 | 50 | 69 | 3 | 20 | 23 |
| A - Acceptance | 17 | 26 | 43 | 5 | 6 | 11 |
| A – Feeling empowered | 18 | 49 | 67 | 4 | 29 | 33 |
| B – Therapist support | 16 | 33 | 49 | 1 | 8 | 9 |
| B – Open to expressing feelings | 2 | 7 | 9 | 0 | 2 | 2 |
| B – No support before ORBIT | 0 | 7 | 7 | 0 | 4 | 4 |
| B – Increased Child/Supporter collaboration | 2 | 15 | 17 | 0 | 2 | 2 |
| C – Struggled to engage | 8 | 37 | 45 | 2 | 22 | 24 |
| C – ORBIT was unclear | 3 | 4 | 7 | 2 | 0 | 2 |
| C – Symptoms increased during ORBIT | 1 | 16 | 17 | 0 | 1 | 1 |
| C – Remaining concerns regarding tics | 39 | 6 | 45 | 2 | 1 | 3 |
| C – Improvement required | 33 | 40 | 73 | 5 | 15 | 20 |
| C – Technical limitations | 2 | 8 | 10 | 0 | 0 | 0 |
| D – No difficulties reported | 6 | 30 | 36 | 0 | 0 | 0 |
| D – No adverse effects reported | 0 | 42 | 42 | 0 | 1 | 1 |
| D – No obvious changes required | 19 | 17 | 36 | 0 | 0 | 0 |
| D – Helpful aspects | 14 | 4 | 18 | 0 | 1 | 1 |
| D – Positive experience of ORBIT | 42 | 42 | 84 | 11 | 19 | 30 |
| D – ORBIT was clear | 4 | 10 | 14 | 0 | 0 | 0 |
| D – Easy to adhere to | 1 | 10 | 11 | 0 | 1 | 1 |
| E – Caused negative feelings | 2 | 27 | 29 | 0 | 10 | 10 |
| E – Interpersonal issues | 2 | 15 | 17 | 0 | 7 | 7 |
| E – Practice was difficult | 16 | 11 | 27 | 0 | 1 | 1 |
| E – No benefit from ORBIT | 4 | 9 | 13 | 0 | 4 | 4 |
| E – Face-to-face (FtF) therapy more suitable | 1 | 6 | 7 | 0 | 1 | 1 |
